# Impact of pre‐existing interstitial lung abnormal shadow on lung injury development and severity in patients of non‐small cell lung cancer treated with osimertinib

**DOI:** 10.1002/cam4.4750

**Published:** 2022-04-17

**Authors:** Ryota Shibaki, Yuichi Ozawa, Susumu Noguchi, Yusuke Murakami, Eri Takase, Yuichiro Azuma, Masaru Maebeya, Takeya Sugimoto, Atsushi Hayata, Takahiro Hayakawa, Shinya Tamaki, Masanori Nakanishi, Shunsuke Teraoka, Hiroaki Akamatsu

**Affiliations:** ^1^ Internal Medicine III Wakayama Medical University Wakayama Japan; ^2^ Respiratory Medicine Japanese Red Cross Wakayama Medical Center Wakayama Japan; ^3^ Respiratory Medicine National Hospital Organization Minami Wakayama Medical Center Wakayama Japan; ^4^ Department of Respiratory Medicine Naga Municipal Hospital Wakayama Japan; ^5^ Respiratory Medicine National Hospital Organization Wakayama Hospital Wakayama Japan; ^6^ Respiratory Medicine Wakayama Rosai Hospital Wakayama Japan; ^7^ Internal Medicine Kinan Hospital Wakayama Japan; ^8^ The Second Department of Internal Medicine Hidaka General Hospital Wakayama Japan

**Keywords:** epidermal growth factor receptor, interstitial lung abnormalities, lung injury, non‐small cell lung cancer

## Abstract

**Background:**

First‐generation epidermal growth factor receptor‐tyrosine kinase inhibitor (EGFR‐TKI) sometimes causes lung injury, thereby affecting survival. Although pre‐existing interstitial lung abnormal shadow (pre‐ILS) increases the risk of lung injury by EGFR‐TKIs, its impact on osimertinib, a third‐generation EGFR‐TKI, remains unknown.

**Patients and Methods:**

This retrospective cohort study consecutively enrolled patients of *EGFR‐*mutated non‐small cell lung cancer treated with osimertinib. Computed tomography images were obtained and evaluated independently by three pulmonologists in a blinded manner. Factors associated with lung injury were assessed using a logistic regression model. Survival curves were calculated by the Kaplan–Meier method and compared using a log‐rank test.

**Results:**

Of the 195 patients, 40 had pre‐ILS, and 21 (8 with and 13 without pre‐ILS) developed lung injury during the observation period. Multivariate analysis revealed that pre‐ILS was independently associated with lung injury (odds ratio, 3.1; 95% confidence interval [CI], 1.1–8.2; *p* = 0.025). Severe (≥Grade 3) lung injury was observed in eight (4.1%) patients, of whom, two (5%) and six (3.9%) had and did not have pre‐ILS (*p* = 0.67), respectively. Grade 5 lung injury was not observed, and survival curves were similar between the patients who developed lung injury and those who did not (median 11 vs. 12 months; hazard ratio, 1.2; 95% CI, 0.56–2.7; *p* = 0.60).

**Conclusions:**

Pre‐ILS increased the risk of lung injury in patients of non‐small cell lung cancer treated with osimertinib, while the severity of lung injury was not clearly affected by the presence of pre‐ILS.

## Graphical abstract

Although pre‐existing interstitial lung abnormal (pre‐ILS) shadow has been known to be a risk factor of lung injury by EGFR‐TKIs, data on osimertinib are lacking. Current results demonstrated that pre‐ILS increases the risk of lung injury (odds ratio, 3.1; 95% confidence interval, 1.1–8.2; *p* = 0.025) in the treatment with osimertinib. Notably, the incidence of grade ≥3 was similar between patients with and without pre‐ILS, which was 5% and 3.9%, respectively. The overall survival was similar for patients who developed lung injury and those who did not. However, we still need to be cautious during treatment with osimertinib in patients with pre‐ILS; further studies are warranted in this field.

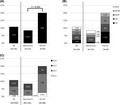

## 1 INTRODUCTION

Osimertinib is the only third‐generation epidermal growth factor receptor‐tyrosine kinase inhibitor (EGFR‐TKI) approved by the Food and Drug Administration, which has led to a breakthrough in the treatment of patients with advanced EGFR‐mutated non‐small cell lung cancer (NSCLC).^1^, ^2^ However, lung injury has been one of the major concerns with the treatment using osimertinib.^3^ First‐ or second‐generation EGFR‐TKIs are shown to cause lung injury in 1.3%–5.3% patients, with a fatality rate as high as 17%–33%.^4^, ^5^ In patients who developed lung injury upon treatment with gefitinib, a first‐generation EGFR‐TKI, the cumulative mortality rate at 12 weeks following treatment initiation was 58.7%, which was 14.6% in the patients who did not develop lung injury.^6^

The presence of pre‐existing interstitial lung abnormal shadow (pre‐ILS) has repeatedly demonstrated the risk of lung injury with systemic chemotherapy, PD‐1/L1 inhibitors,^6^, ^7^, ^8^ or EGFR‐TKIs^6^, ^9^; the incidence of the lung injury in patients with pre‐ILS who received gefitinib was as high as 14%–33%.^9^, ^10^ However, the impact of pre‐ILS on the treatment with osimertinib is yet to be elucidated.

Thus, this study aimed to identify the risk factors of lung injury in the treatment with osimertinib, with a focus on chest image characteristics.

## 2 METHODS

### 2.1 Patients and ethical considerations

This study was conducted in accordance with the provisions of the Declaration of Helsinki and was approved by the Ethics Committee of Wakayama Medical University (No. 2172). To avoid case selection bias, we collected all the cases consecutively in which osimertinib was prescribed in Wakayama Prefecture between November 2015 (when osimertinib was approved in Japan) and June 2019. Specifically, we contacted all the hospitals that treat lung cancer in Wakayama Prefecture and requested eight institutions with at least one case of osimertinib prescription to participate in this study. We obtained consent from all the institutions. The median observation period was 12 months (range, 0.4–44 months).

### 2.2 Evaluation of computed tomography findings

Interstitial lung abnormal shadow was defined as the presence of nondependent changes, which included ground‐glass or reticular abnormalities, diffuse centrilobular nodularity, non‐emphysematous cysts, honeycombing, and traction bronchiectasis regardless of the lung field occupying area. The lung area affected by pre‐ILS was classified into three grades based on the chest computed tomography (CT) findings as follows: 0%, 1%–4%, and ≥5%. In line with a position paper from the Fleischner Society in 2020, interstitial lung abnormalities (ILA) were defined as the occupation of more than 5% of the lung field among the ILS, and the others (the occupation of less than 5% of the lung field) was assigned to indeterminant‐ILA (ind‐ILA). Radiation pneumonitis, which was determined according to the history of radiation therapy and imaging features, and shadow by lung cancer were excluded from all image evaluations. Computed tomography images were reconstructed using a 1–5‐mm slice thickness. Three pulmonologists (Y.O., A.H., and M.N.) independently performed a review of the serial CT images. All evaluations were completed without any preliminary knowledge of the patients or other doctors' decisions. Final decisions were made based on a majority, and when the results were equally divided, a consensus was made through discussion. These methods for the chest image evaluation were previously used and reported.^8^, ^11^, ^12^

### 2.3 Diagnosis and radiographic patterns of osimertinib‐induced lung injury

The diagnostic criteria for osimertinib‐induced lung injury were as follows: (1) newly emerged lesion during treatment with osimertinib, (2) ground‐glass attenuation or infiltrative shadow on chest CT, (3) no indication of pulmonary infection including purulent sputum, improvement by antibiotic treatment, and positive results for sputum and/or blood cultures. Patients with an abnormal shadow accompanied by a relevant bronchial obstruction, apparent heart failure, or pulmonary invasion of lung cancer were excluded. In cases of lung injury, chest CT images of the lung injury were also evaluated by three pulmonologists as described above. The phenotypical patterns of lung injury were classified as acute eosinophilic pneumonia (AEP), non‐cardiogenic pulmonary edema (NCPE), non‐specific interstitial pneumonia (NSIP), organizing pneumonia (OP), hypersensitivity pneumonia (HP), or diffuse alveolar damage (DAD) according to the consensus statement for the diagnosis and treatment of drug‐induced lung injuries from Japanese Respiratory Society, which was based on an official American Thoracic Society/European Respiratory Society statement for the international multidisciplinary classification of the idiopathic interstitial pneumonias.^13^, ^14^

### 2.4 Statistical analysis

Overall survival was defined as the interval from the date of initial administration of osimertinib to the date of death from any cause or of the last follow‐up examination. Toxicity was assessed using the Common Terminology Criteria for Adverse Events version 5.0.

Dichotomous variables, such as baseline characteristics, are expressed in numbers and percentages and were compared using Fisher's exact test. Continuous variables are represented as median values and interquartile ranges and were analyzed using the Wilcoxon rank‐sum test. The curves of the cumulative incidence of osimertinib‐induced lung injury were constructed using the Kaplan–Meier method. Multivariate analysis was performed for the factors with *p* < 0.2 in the univariate analysis. All *p*‐values were based on a two‐sided hypothesis, and *p* < 0.05 was considered statistically significant. All the statistical analyses were performed using JMP Pro software version 14.1.0 (SAS Institute).

## 3 RESULTS

### 3.1 Patient characteristics

This study included 195 patients with advanced or recurrent *EGFR*‐mutated NSCLC treated with osimertinib. Baseline characteristics are shown in Table 1. The median age was 73 years, and 45% of the participants were ≥75 years old. Additionally, 158 patients (81%) had a performance status of 0 or 1 when osimertinib treatment was initiated. The *EGFR* mutations were distributed as follows: exon 19 deletion in 41 patients (21%), L858R in 37 patients (19%), major mutation plus T790M in 113 patients (58%), and other mutations in four patients (2.1%). Finally, 115 patients (59%) had received previous EGFR‐TKI treatment, and one patient had a history of immune‐checkpoint inhibitor (ICI) treatment.

**TABLE 1:** Baseline characteristics

	All, *n* (%)	Preexisting ILS		*p*
		Yes, *n* (%)	No, *n* (%)	
Patients	195	40	155	
Age				
Median (range)	73 (44–95)	77 (46–88)	72 (44–95)	0.0043
≥75 years	87 (45)	26 (65)	61 (39)	
Sex				0.59
Male	71 (36)	16 (40)	55 (35)	
ECOG PS				0.27
0–1	158 (81)	30 (75)	128 (83)	
≥2	37 (19)	10 (25)	27 (17)	
Smoking status				0.28
Never‐smoker	117 (60)	21 (53)	96 (62)	
Current or former smoker	78 (40)	19 (47)	59 (38)	
EGFR mutation type				
Major mutation	78 (40)	11 (28)	67 (43)	
Major + T790M	113 (58)	27 (68)	86 (55)	
Other	4 (2.1)	2 (5)	2 (1.3)	
EGFR‐TKI				0.15
Naïve	80 (41)	12 (30)	68 (44)	
Treated	115 (59)	28 (70)	87 (56)	
Thoracic radiotherapy	6 (3.1)	4 (10)	2 (1.3)	0.017
Previous treatment with ICIs	1 (0.51)	0 (0.0)	1 (0.65)	
Preexisting ILS				
Indeterminant ILA		19 (48)		
ILA		21 (53)		

Abbreviations: ECOG PS, Eastern Cooperative Oncology Group; performance status; EGFR, epidermal growth factor receptor: major mutation, exon 19 deletion and L858R mutation; ICIs, immune checkpoint inhibitor; ILA, interstitial lung abnormalities; ILS, interstitial lung abnormal shadow; TKI, tyrosine kinase inhibitor.

Based on the central review of chest CT images immediately before administration of osimertinib, 40 patients (21%) were determined to have pre‐ILS, of which 21 cases were classified as pre‐ILA and 19 as pre‐ind‐ILA. A honeycomb pattern was detected in only one patient. Patients with pre‐ILS tended to be older (median age [range], 77 [46–88] vs. 72 [44–95] years; *p* = 0.0043) and have a higher rate of prior thoracic radiotherapy (10% vs. 1.3%; *p* = 0.017) than those without pre‐ILS. The remaining observed factors were similar in both groups.

### 3.2 Predictive factors of lung injury

**FIGURE 1:**
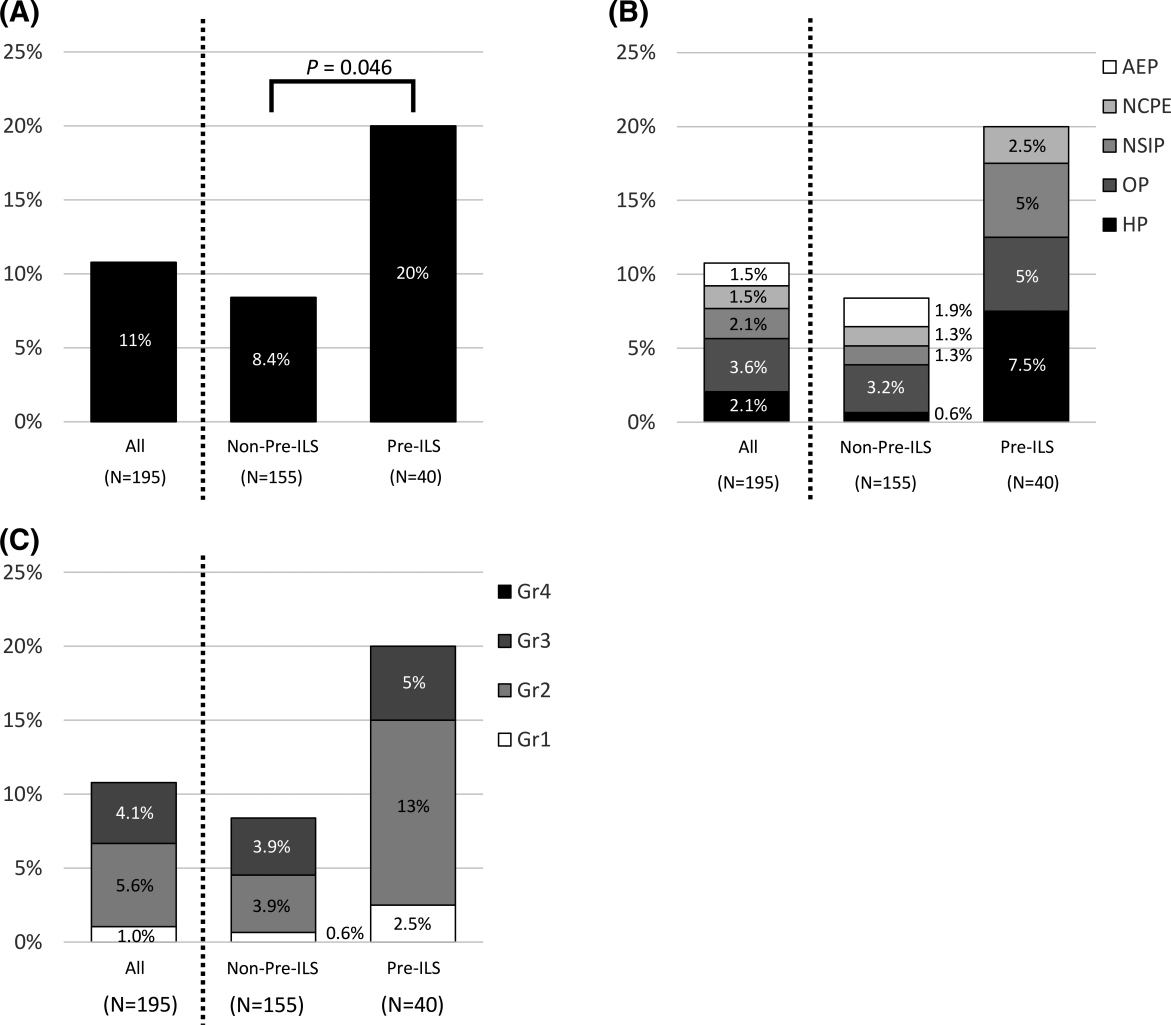
The incidence of lung injury. Comparison of (A) incidence, (B) radiographic patterns, and (C) severity of lung injury in patients with and without pre‐existing interstitial lung abnormal shadow. AEP, acute eosinophilic pneumonia; Gr, grade; HP, hypersensitivity pneumonia; NCPE, non‐cardiogenic pulmonary edema; NSIP, non‐specific interstitial pneumonia; OP, organizing pneumonia; pre‐ILS, pre‐existing interstitial lung abnormal shadow

Twenty‐one patients (11%) developed lung injury during the observation period. The incidence of lung injury was significantly higher among patients with pre‐ILS than among those without pre‐ILS (20% vs. 8.4%; *p* = 0.046) (Figure 1A). Moreover, the incidence of lung injury was 14% and 26% in patients with pre‐ILA and pre‐ind‐ILA, respectively (*p* = 0.44) (Figure S1). To explore the predictive factors of osimertinib‐induced lung injury, we first performed a univariate analysis of the multiple factors (Table 2). We found that the presence of pre‐ILS was significantly associated with the development of osimertinib‐induced lung injury. According to the multivariate analysis adjusted for performance status, pre‐ILS was the only significant predictive factor of lung injury (odds ratio, 3.1; 95% confidence interval [CI], 1.1–8.2; *p* = 0.025).

**TABLE 2:** Cox‐proportional hazard regression analyses

	Univariate analysis		Multivariate analysis	
	Odds ratio (95% CI)	*p*	Odds ratio (95% CI)	*p*
Pre‐ILS (yes/no)	2.7 (1.0–7.1)	0.040	3.1 (1.1–8.2)	0.025
Pre‐ILA (yes/no)	1.4 (0.39–5.4)	0.58		
Age (≥75 years/<75 years)	1.4 (0.57–3.5)	0.45		
Sex (female/male)	1.2 (0.45–3.0)	0.76		
ECOG PS (0–1/≥2)	5.2 (0.68–40)	0.11	6.0 (0.77–47)	0.088
Smoking status (current or former/never)	1.1 (0.46–2.9)	0.78		
EGFR‐TKI (naive/treated)	1.1 (0.44–2.7)	0.86		
%VC (≥80%/<80%)	1.9 (0.40–9.1)	0.42		

Abbreviations: CI, confidence interval; HR, hazard ratio; ECOG PS, Eastern Cooperative Oncology Group performance status; EGFR‐TKI, epidermal growth factor receptor tyrosine kinase inhibitor; ILA, interstitial lung abnormalities; ILS, interstitial lung abnormal shadow; PFS, progression‐free survival; VC, vital capacity.

### 3.3 Severity and radiographic patterns of lung injury

The distribution of lung injury severity was as follows: grade 1 in two patients (1.0%), grade 2 in 11 patients (5.6%), and grade 3 in eight patients (4.1%). The incidence of severe lung injury (Common Terminology Criteria for Adverse Events Grade ≥3) was 5% and 3.9% in patients with or without pre‐ILS, respectively (*p* = 0.67), and none of the patients developed Grade 5 lung injury (Figure 1C). One patient in the pre‐ILA group and one in the ind‐ILA group developed severe pneumonitis (Figure S1C).

Considering the radiographic patterns of lung injury, OP was the most common pattern, observed in seven patients (3.6%), followed by HP and NSIP in four patients each (2.1%), and AEP and NCPE in three patients each (1.5%). Diffuse alveolar damage pattern was not observed. The distribution of the radiographic patterns of lung injury was similar between patients with and without pre‐ILS (Figure 1B) and in patients with pre‐ILA and ind‐ILA (Figure S1A). Of the 21 patients who developed lung injury, 19 (90%) received steroid therapy and 2 (9.5%) exhibited improved clinical and radiographic outcomes without immunosuppression therapy. Osimertinib was not readministered in any of the patients.

### 3.4 Cumulative incidence of lung injury

**FIGURE 2:**
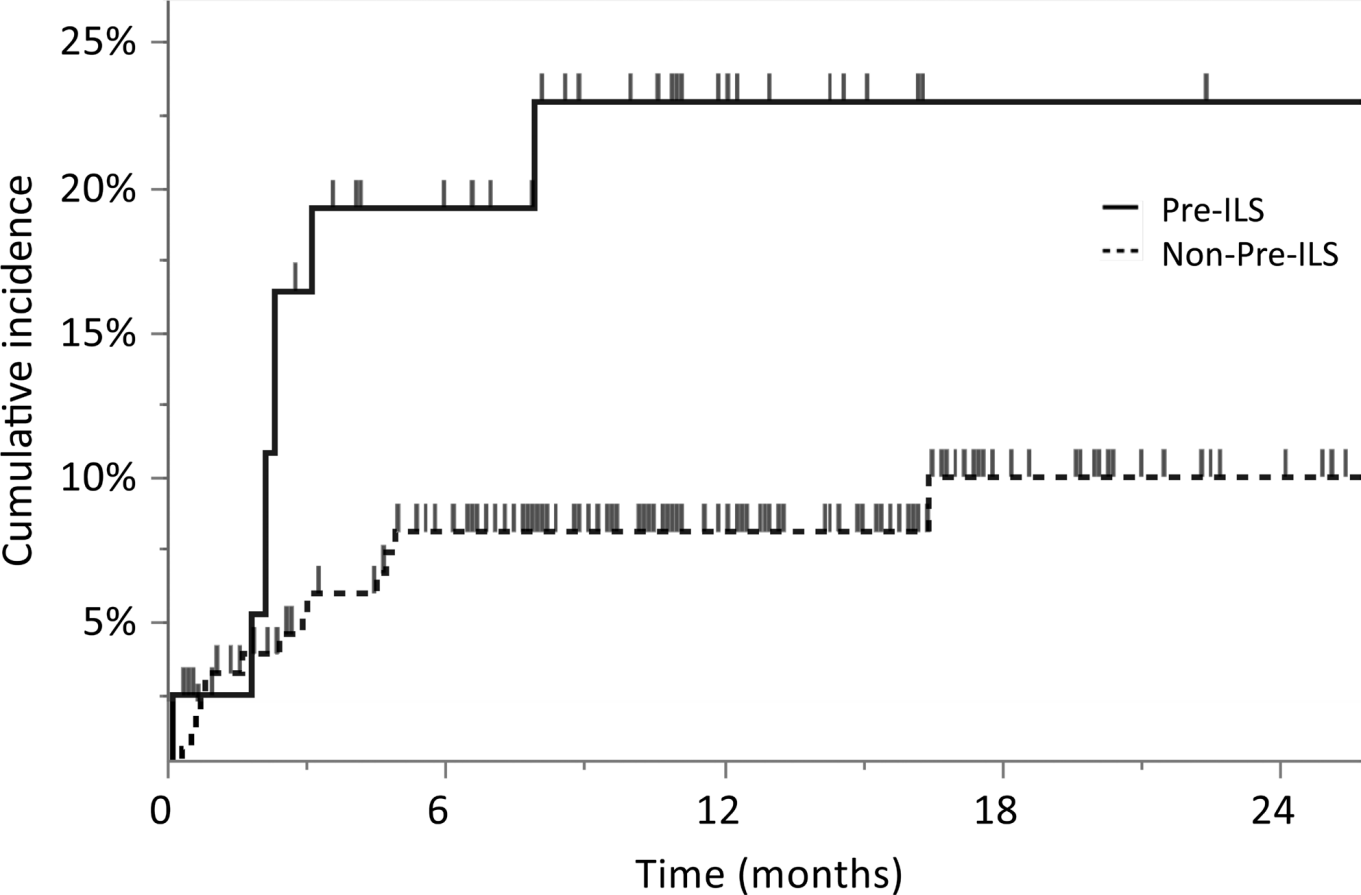
Cumulative incidence of lung injury. Kaplan–Meier curves for cumulative incidence of osimertinib‐induced lung injury were stratified by patients with (*n* = 40) and without (*n* = 155) pre‐existing interstitial lung abnormal shadow. The vertical bars indicate censored. Pre‐ILS, pre‐existing interstitial lung abnormal shadow

During the observation period, the median interval from the initiation of osimertinib treatment to the onset of pneumonitis was 2.2 months (range, 0.1–7.9 months) and 2.4 months (range, 0.3–16.4 months) in patients with and without pre‐ILS, respectively. The incidence of pneumonitis at 1 month was 16% (95% CI, 7.6–32) in patients with pre‐ILS and 6.0% (95% CI, 3.2–11) in patients without pre‐ILS; at 3 months, which increased to 19% (95% CI, 9.5–35) and 8.2% (95% CI, 4.7–14), respectively (Figure 2). The cumulative incidence of pneumonitis, as determined by the Kaplan–Meier method, was higher in patients with pre‐ILS than in those without pre‐ILS (hazard ratio [HR], 2.9; 95% CI, 1.2–7.0; *p* = 0.014).

### 3.5 Impact of lung injury on survival

**FIGURE 3:**
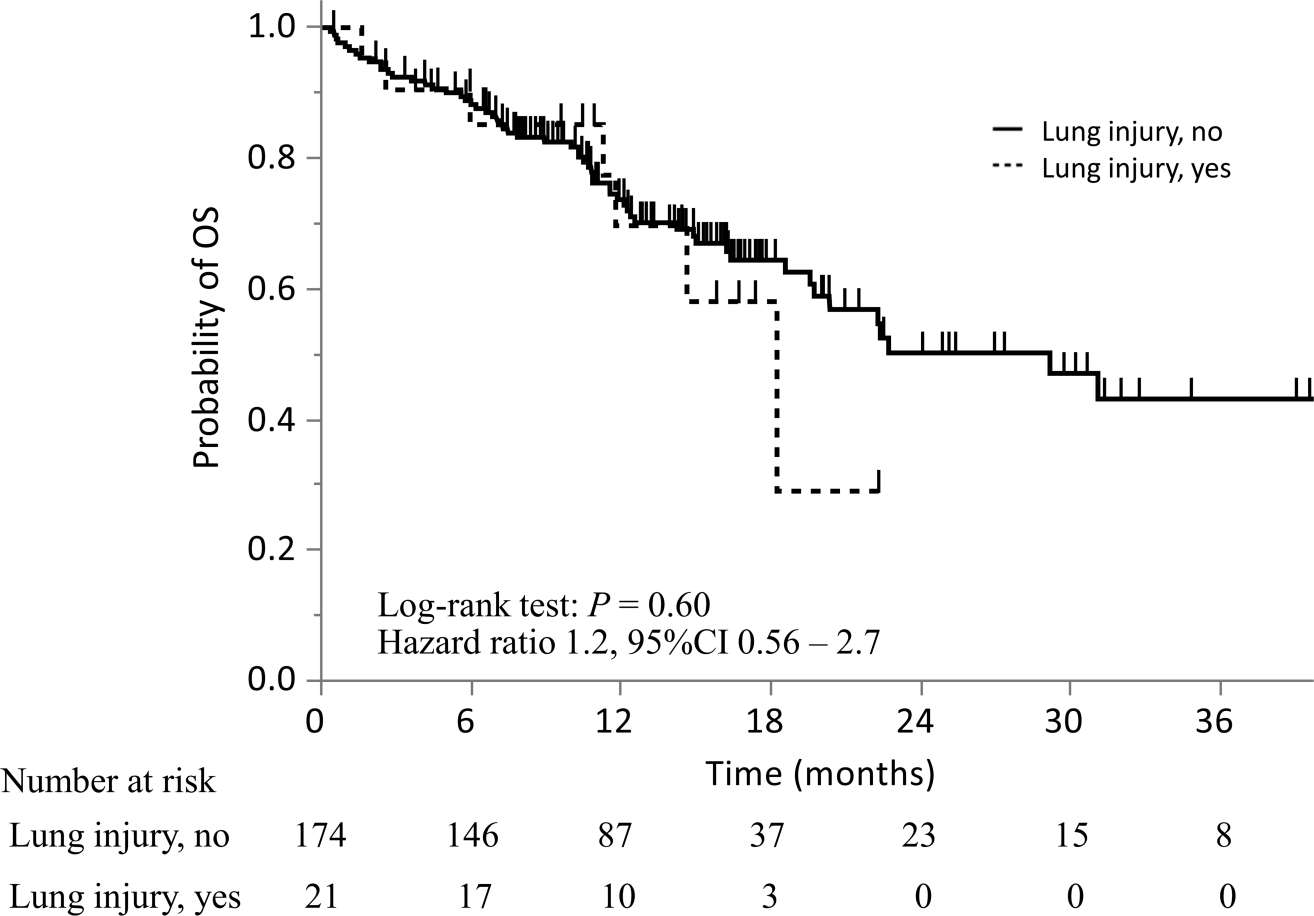
Overall survival of patients classified based on the development of lung injury. Kaplan–Meier curves for overall survival were stratified by patients who did (*n* = 21) and did not (*n* = 174) develop lung injury. CI, confidence interval; OS, overall survival

To examine the relationship between lung injury and survival, we created and subsequently compared the survival curves for patients who developed lung injury and those who did not (Figure 3). At the time of data cutoff, 65 patients had died: 7 of the 21 patients (33%) who developed lung injury and 58 of the 174 patients (33%) who did not. In patients who did or did not develop lung injury, the survival curves were similar, and the median survival time from starting osimertinib was 11 months (range, 1.6–22 months) and 12 months (range, 0.36–44 months), respectively (HR, 1.2; 95% CI, 0.56–2.7; *p* = 0.60). Patient characteristics did not significantly differ between the two groups (Table S1).

## 4 DISCUSSION

The current study reports two novel results regarding the association between pre‐ILS (pre‐ILA and pre‐ind‐ILA) and osimertinib‐induced lung injury centrally evaluated on chest CT images of patients treated with osimertinib. First, pre‐ILS was identified as an independent risk factor for the development of osimertinib‐induced lung injury. Second, the severity of lung injury was similar between the patients with and without pre‐ILS.

A recent report summarizing a post‐marketing survey demonstrated that the presence of pre‐ILS and a history of nivolumab treatment are risk factors for the development of osimertinib‐induced lung injury.^3^ The frequency of pre‐ILS in that report (2.9%) was notably lower than that in previous studies.^15^, ^16^ This may have been due to the nature of the survey, in which the presence of pre‐ILS was determined solely by the judgment of the attending physician without a clear definition; thus, cases with minor interstitial shadows were often overlooked. Importantly, pre‐ILA was detected in 10.8% of all cases in that study, which was comparable to that in previous studies (10%–14%),^15^, ^16^ proving that the central imaging review conducted in the current study was reliable and in line with the general criteria. The frequency of lung injury was 11% in this report, which was higher than the frequency in the above‐mentioned post‐marketing surveillance report, 6.8%. This may be because 41% of patients received osimertinib as first‐line therapy in this study, while 97.6% of patients in the previous report had a history of treatment with EGFR‐TKI,^3^ which means patients who developed lung injury or died from the first EGFR‐TKI were excluded. Another factor might be the different pre‐ILS ratios.

The present results are the first to demonstrate that the presence of pre‐ILS is a risk factor for osimertinib‐induced lung injury. This study's definition of ILS includes both ILA and ind‐ILA. Since ind‐ILA accounted for 48% of pre‐ILS, it is likely that the cases with relatively minor shadows were accounted for. Notably, the current results also suggest that the extent of pre‐ILS is not critical for the risk of lung injury by osimertinib.

Furthermore, we also demonstrated that the presence of pre‐ILS does not affect the severity of osimertinib‐induced lung injury. In patients with pre‐ILS, the severity of lung injury caused by ICIs or chemotherapy is often high. In our previous report of lung injury caused by docetaxel or S‐1 in patients with pre‐ILS, 14 of 18 lung injuries (78%) were Grade 3 or higher.^8^ In addition, phase II study of atezolizumab in patients of NSCLC with idiopathic interstitial pneumonia revealed that four of the five patients (80%) who developed lung injury were Grade 3 or higher and one was Grade 5.^17^ Few reports exist on the effect of pre‐ILS on the severity of lung injury induced by EGFR‐TKIs. Our current results, together with the fact that there were no deaths in patients with or without pre‐ILS, suggest the need to further investigate the clinical significance of pre‐ILS in treatment with osimertinib.

In gefitinib treatment, with approximately 59.9% of severe cases and 35.7%–44.3% of fatal cases, 15–24% of the gefitinib‐induced lung injuries demonstrated DAD patterns in the chest CT images.^18^, ^19^ In contrast, the current real‐world study demonstrated severe cases at a rate of 38.1%, and no death related to lung injuries or DAD pattern was observed. These results are congruent with the results from previous phase III trials^1^, ^2^ (severe cases: 10%–50%, fatal cases: 0%–10%), and a recent retrospective study by Kodama et al.^20^ (severe cases: 25%, fatal cases: 3.6%, DAD patterns: 7.1%). The less frequent DAD patterns in osimertinib‐induced lung injury may result in its weaker impact on survival than that of gefitinib‐induced lung injury.

The mechanisms underlying drug‐induced lung injury are thought to involve direct drug‐induced damage to type II alveolar epithelial cells and activation of immune cells. Gefitinib has been reported to increase the vulnerability of epithelial cells caused by suppression of Hsp70 expression, suggesting that EGFR‐TKIs may damage epithelial cells by their action on normal alveolar epithelium.^21^ Therefore, EGFR‐TKI‐induced lung injury causes irreversible lung damage, resistance to steroid therapy, and a high mortality rate.^6^, ^9^, ^19^ Although there is little preclinical data regarding osimertinib‐induced lung injury, it reportedly responds well to steroid treatment and is often reversible in clinical practice.^22^, ^23^ Similarly, our study demonstrated that 19 of 21 patients with lung injuries received corticosteroids and survived. The difference in response to steroids between patients treated with first‐generation EGFR‐TKIs and those treated with osimertinib indicates different mechanisms of lung injury induced by each drug, which may also explain the differences in their impact on survival.

This study had certain limitations. First, we evaluated patients based on a retrospective review of the medical records from multiple hospitals. Inevitably, there are inter‐hospital differences in CT slice thickness, CT interval, and patient selection for osimertinib treatment. In addition, the diagnosis and treatment of lung injury were based on the criteria that excluded pulmonary infection and heart failure; however, the final decision of diagnosis was made by physicians, allowing for potential bias. Moreover, there was only one case of a honeycomb pattern in this study. This indicates that cases with obvious pre‐ILS may have been excluded from the study because previous reports showed that EGFR‐TKI‐induced lung injury is more common in patients with pre‐ILS. Therefore, the results of the current study, which showed that the severity of lung injury and imaging patterns did not change with the presence of pre‐ILS, should not be immediately applied to all cases of pre‐ILS, especially in patients with honeycomb patterns. Caution should be exercised in these cases until further investigation is performed.

Nevertheless, this study is one of the largest investigations focusing on lung injury in patients treated with osimertinib, and all pre‐treatment chest CT images were independently evaluated in a blinded manner. Consequently, the overall bias was relatively low since all the patients treated with osimertinib in Wakayama Prefecture were included.

### 4.1 Conclusions

The presence of pre‐ILS increases the risk of lung injury in patients of NSCLC treated with osimertinib. Although the severity of lung injury was not clearly affected by pre‐ILS and the impact of osimertinib‐induced lung injury on survival could be weak, we still need to be cautious during treatment with osimertinib in patients with pre‐ILS.

## CONFLICT OF INTEREST

Dr. Ozawa reported receiving honoraria from AstraZeneca K.K. and Chugai Pharmaceutical Co. Ltd. and participation on an advisory board for AstraZeneca K.K. Dr. Teraoka reported receiving consulting fees from Pfizer Inc. and honoraria from AstraZeneca K.K., Boehringer Ingelheim Japan Inc., Chugai Pharmaceutical Co. Ltd., Eli Lilly Japan K.K., Novartis Pharma K.K., Ono Pharmaceutical Co. Ltd., and Taiho Pharmaceutical Co. Ltd. Dr. Akamatsu reported receiving honoraria from AstraZeneca K.K., Boehringer Ingelheim Japan Inc., Bristol‐Myers Squibb, Chugai Pharmaceutical Co. Ltd., Eli Lilly Japan K.K., MSD K.K., Novartis Pharma K.K., Ono Pharmaceutical Co. Ltd., and Taiho Pharmaceutical Co. Ltd. and research funding from Chugai Pharmaceutical Co. Ltd. and MSD K.K. The remaining authors declare no conflict of interest.

## AUTHOR CONTRIBUTIONS

**Ryota Shibaki:** Data curation; methodology; formal analysis; investigation; resources; visualization; project administration; writing – original draft; and writing – review and editing. **Yuichi Ozawa:** Investigation; validation; writing – original draft; and writing – review and editing. **Susumu Noguchi:** Investigation; resources; and writing – review and editing. **Yusuke Murakami:** Investigation; resources; and writing – review and editing. **Eri Takase:** Investigation; resources; and writing – review and editing. **Yuichiro Azuma:** Investigation; Resources; and writing – review and editing. **Masaru Maebeya:** Investigation; resources; and writing – review and editing. **Takeya Sugimoto:** Investigation; resources; and writing – review and editing. **Atsushi Hayata:** Investigation; validation; resources; and writing – review and editing. **Takahiro Hayakawa:** Investigation; resources; and writing – review and editing. **Shinya Tamaki:** Investigation; resources; and writing – review and editing. **Masanori Nakanishi:** Investigation; validation; resources; and writing – review and editing. **Shunsuke Teraoka:** Investigation; resources; and writing – review and editing. **Hiroaki Akamatsu:** Investigation; resources; and writing – review and editing.

## ETHICAL APPROVAL STATEMENT

The study design was approved by the appropriate ethics review board of the Wakayama Medical University, Wakayama, Japan (No. 2172). This study was conducted in accordance with the principles of the Declaration of Helsinki. We did not obtain formal consent from the patients because of the nature of the retrospective observational study. Instead, we provided all patients with the opportunity to opt out.

## Supporting information


Figure S1

Table S1

## Data Availability

The data that support the findings of this study are available on request from the corresponding author. The data are not publicly available due to privacy or ethical restrictions.
