# Supplementary figures and images for: Impact of pre‐existing interstitial lung abnormal shadow on lung injury development and severity in patients of non‐small cell lung cancer treated with osimertinib
Source: Cancer Med. 2022 Apr 17;11(20):3743–50. doi: 10.1002/cam4.4750 (PMC9582680; doi:10.1002/cam4.4750)

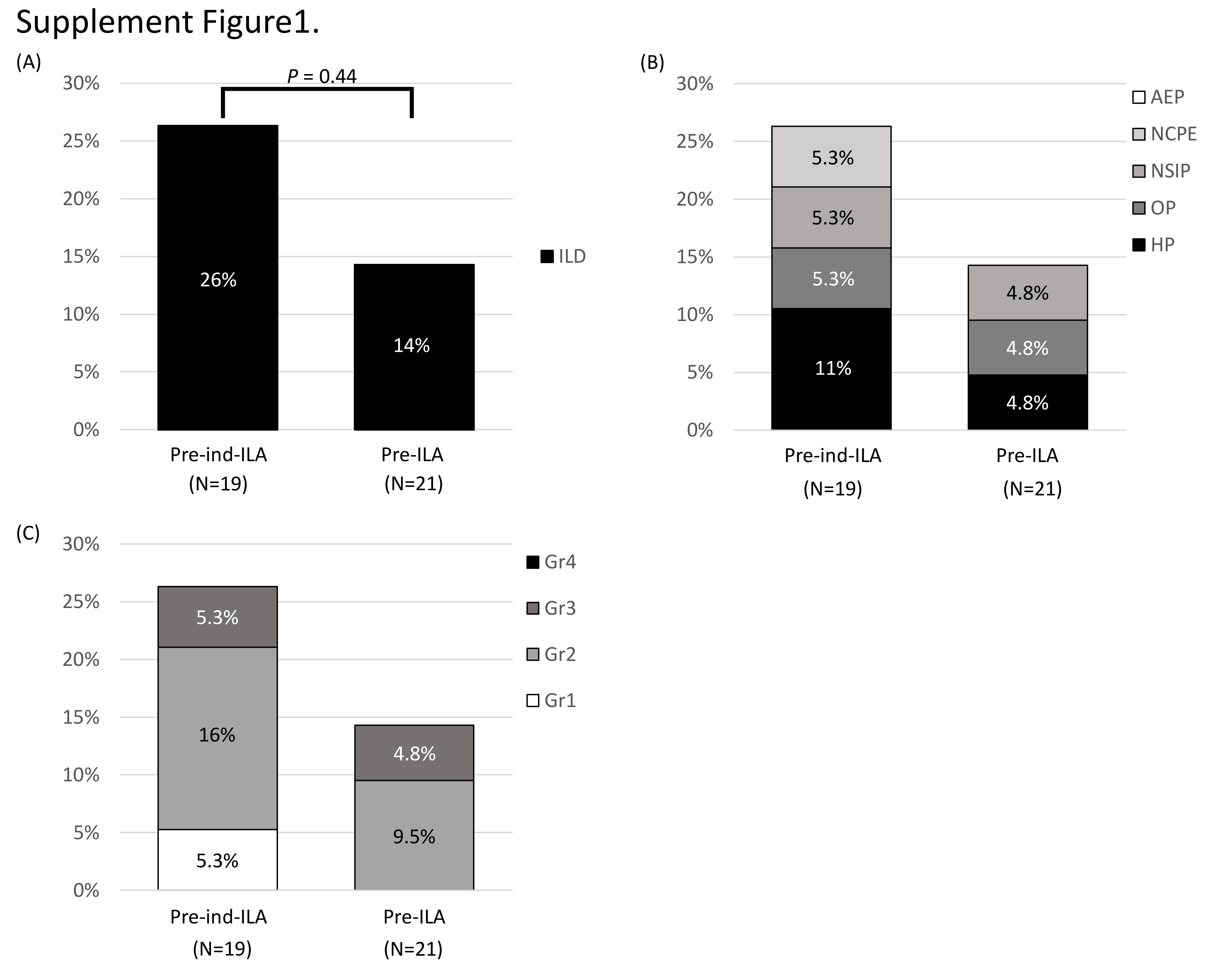

Supplement: Supplementary file 1 — Figure S1 [file CAM4-11-3743-s002.tif]
