# Supplementary material for: Impact of pre‐existing interstitial lung abnormal shadow on lung injury development and severity in patients of non‐small cell lung cancer treated with osimertinib
Source: Cancer Med. 2022 Apr 17;11(20):3743–50. doi: 10.1002/cam4.4750 (PMC9582680; doi:10.1002/cam4.4750)
Supplement: Supplementary file 2 — Table S1 [file CAM4-11-3743-s001.docx]

**Supporting Information**

**Table S 1.** Baseline characteristics

|  | Lung injury | | *p* |
| --- | --- | --- | --- |
|  | Yes, n (%) | No, n (%) |  |
| Patients | 21 | 174 |  |
| Age |  |  | 0.21 |
| median (range) | 76 (55–85) | 73 (44–95) |  |
| ≥ 75 years | 11 (52) | 76 (44) |  |
| Sex |  |  | 0.82 |
| Male | 7 (33) | 64 (37) |  |
| ECOG PS |  |  | 0.14 |
| 0–1 | 20 (95) | 138 (79) |  |
| ≥2 | 1 (4.8) | 36 (21) |  |
| Smoking status |  |  | 0.82 |
| Never-smoker | 12 (57) | 105 (60) |  |
| Current or former smoker | 9 (43) | 69 (40) |  |
| EGFR mutation type |  |  |  |
| Major mutation | 9 (43) | 69 (40) |  |
| Major + T790M | 12 (57) | 101 (58) |  |
| Other | 0 (0.0) | 4 (2.3) |  |
| EGFR-TKI |  |  | 1.0 |
| Naive | 9 (43) | 71 (41) |  |
| Treated | 12 (57) | 103 (59) |  |
| Thoracic radiotherapy | 0 (0.0) | 6 (3.4) | 1.0 |
| Previous treatment with ICIs | 1 (4.8) | 0 (0.0) | 0.33 |
| Preexisting ILS |  |  |  |
| Indeterminant ILA | 5 (24) | 14 (8.0) |  |
| ILA | 3 (14) | 18 (10) |  |

ECOG PS, Eastern Cooperative Oncology Group; performance status; EGFR, Epidermal Growth Factor Receptor: Major mutation, exon 19 deletion and L858R mutation: ICIs, Immune Checkpoint Inhibitor; ILS, interstitial lung abnormal shadow; ILA, interstitial lung abnormalities.

**Figure S1.** Incidence of lung injury. Comparison of (**A**) incidence, (**B**) radiographic patterns, and (**C**) severity of lung injury in patients with pre-existing indeterminant interstitial lung abnormalities and determinant interstitial lung abnormalities.

Pre, pre-existing; ind, indeterminant; ILA, interstitial lung abnormalities; AEP, acute eosinophilic pneumonia; NCPE, non-cardiogenic pulmonary edema; NSIP, non-specific interstitial pneumonia; OP, organizing pneumonia; HP, hypersensitivity pneumonia; Gr, Grade
